# Members of the paralogous gene family 12 from the Lyme disease agent *Borrelia burgdorferi* are non-specific DNA-binding proteins

**DOI:** 10.1371/journal.pone.0296127

**Published:** 2024-04-16

**Authors:** Kalvis Brangulis, Inara Akopjana, Laura Drunka, Sofija Matisone, Diana Zelencova-Gopejenko, Shapla Bhattacharya, Janis Bogans, Kaspars Tars

**Affiliations:** 1 Latvian Biomedical Research and Study Centre, Riga, Latvia; 2 Latvian Institute of Organic Synthesis, Riga, Latvia; University of Kentucky College of Medicine, UNITED STATES

## Abstract

Lyme disease is the most prevalent vector-borne infectious disease in Europe and the USA. *Borrelia burgdorferi*, as the causative agent of Lyme disease, is transmitted to the mammalian host during the tick blood meal. To adapt to the different encountered environments, *Borrelia* has adjusted the expression pattern of various, mostly outer surface proteins. The function of most *B*. *burgdorferi* outer surface proteins remains unknown. We determined the crystal structure of a previously uncharacterized *B*. *burgdorferi* outer surface protein BBK01, known to belong to the paralogous gene family 12 (PFam12) as one of its five members. PFam12 members are shown to be upregulated as the tick starts its blood meal. Structural analysis of BBK01 revealed similarity to the coiled coil domain of structural maintenance of chromosomes (SMC) protein family members, while functional studies indicated that all PFam12 members are non-specific DNA-binding proteins. The residues involved in DNA binding were identified and probed by site-directed mutagenesis. The combination of SMC-like proteins being attached to the outer membrane and exposed to the environment or located in the periplasm, as observed in the case of PFam12 members, and displaying the ability to bind DNA, represents a unique feature previously not observed in bacteria.

## Introduction

*Borrelia burgdorferi* is the causative agent of Lyme disease, as the spirochete can infect the mammalian host after a bite of an infected *Ixodes* tick [1, 2]. Currently, more than a dozen different *Borrelia* species have been described and assembled into the *B*. *burgdorferi* sensu lato complex, of which *B*. *burgdorferi* sensu stricto (hereafter *B*. *burgdorferi*), *B*. *garinii*, *B*. *afzelii*, *B*. *spielmanii*, *B*. *mayonii*, and *B*. *bavariensis* are associated with Lyme disease [3–5]. *B*. *burgdorferi* is the major pathogenic genospecies geographically associated with North America, while *B*. *garinii*, *B*. *afzelii*, *B*. *spielmanii*, and *B*. *bavariensis* are associated with Europe [3]. Interestingly, different *Borrelia* species can cause distinct clinical manifestations, as *B*. *burgdorferi* is strongly related to Lyme arthritis, while *B*. *garinii* and *B*. *afzelii*, the most prevalent *Borrelia* species in Europe, are associated with neurological or skin manifestations, respectively [3]. Regardless of the pathogenic *Borrelia* species located in the infected tick, once the bacteria from the tick gut enter the mammalian host, they must be able to fight against the host’s immune system, attach to different host cells, and disseminate [6]. Therefore, to adjust to the specific requirements, *Borrelia* has managed to change the expression levels of various proteins depending on the environmental conditions [7–10]. To successfully infect the new host, surface-exposed outer surface lipoproteins potentially play the most important role, as they interact with the environment. In comparison to any other gram-negative bacteria, *B*. *burgdorferi* is exceptionally rich in outer surface proteins that are covalently attached to the cell membrane by an N-terminal lipid modification and are therefore called lipoproteins [11–13]. In *B*. *burgdorferi*, there are at least 140 predicted lipoprotein genes, of which approximately 100 are upregulated once the tick starts its blood meal [8, 13–15]. Unfortunately, the exact function is known for only a small fraction of all the predicted lipoproteins. There are several lipoproteins, for example, BBA07, BBA64, BBA57, BBE31, BB0238, and BB0365 that are known to be important in the pathogenesis of Lyme disease, although the exact function, ligand, or receptor has not yet been determined [16–21]. For the majority of outer surface proteins, it is impossible to predict the function solely by sequence comparison, as no homologous proteins can be found in any other organism outside the genus *Borrelia* [22]. However, most of the protein-coding genes within Lyme disease causing *Borrelia* have at least one paralogous gene; thus, as a whole, there are approximately 160 paralogous gene families (PFams) in *B*. *burgdorferi* [13]. In the current study we focused on the paralogous gene family 12 (PFam12), which contains five members (BB0844, BBG01, BBH37, BBJ08, and BBK01) that do not demonstrate meaningful sequence identity with proteins from any other organism which would allow us to judge the functions of these proteins. It is known that PFam12 members are surface-exposed and highly immunogenic lipoproteins that are upregulated as the tick starts the blood meal [8, 13, 14, 23–27]. In a recent article studying the antigenicity and immunogenicity of PFam12 members, it was suggested to rename the proteins as family twelve lipoproteins (Ftl) FtlA (BBK01), FtlB (BBG01), FtlC (BBH37), and FtlD (BBJ08) [27].

Our data demonstrate that PFam12 members are non-specific DNA-binding proteins. These results not only identify a putative ligand for all five PFam12 member proteins, but also reveal the unique localization of SMC-like proteins on the bacterial surface and periplasm. Understanding the role of this interaction could enhance our understanding of Lyme disease pathogenesis, potentially leading to the development of new strategies to fight against Lyme disease. Additionally, given the similarity between the members of PFam12 as described below, we suggest that BB0844, as a member of PFam12, should be called FtlE.

## Materials and methods

### Cloning and expression of PFam12 members

The same procedure was used for all PFam12 member proteins (FtlA, FtlB, FtlC, FtlD, and FtlE). Before cloning, the signal sequence regions were predicted using SignalP 4.1 [28]. PFam12 members were PCR amplified from *B*. *burgdorferi* strain B31 by excluding the signal sequence. The amplified genes were cloned into the pETm-11 expression vector containing a 6xHis tag and TEV protease recognition site. The recombinant pETm-11 plasmids encoding FtlA_27-297_, FtlB_27-297_, FtlC_35-312_, FtlD_126-306_ FtlE_95-323_ and were transformed into *Escherichia coli* XL1-Blue cells, incubated on LB agar plates at 37°C for 24 h, and then separate colonies were cultivated in LB medium prior to isolation of the plasmid DNA. Correct constructs as judged from the DNA sequencing were transformed and expressed in *E*. *coli* BL21 (DE3). The expression was induced by the addition of 0.2 mM IPTG and carried out in 2xTY media at 37°C for 8 h. Production of N-terminally truncated PFam12 proteins FtlA_110-297_, FtlB_110-297_, FtlC_131-312_, FtlD_126-306_, and FtlE_95-323_ FtlA_110-297_ mutant proteins was carried out as described above. The expression of Se-Met-labeled FtlA was performed as previously described [29].

### Protein purification and 6xHis tag cleavage

*E*. *coli* cells were lysed by sonication, and the lysate soluble fraction after centrifugation at 10 000 rpm for 30 min was transferred to a Ni-NTA agarose (Protino) column. The eluted protein fraction was concentrated and buffer exchanged into 20 mM Tris-HCl (pH 8.0) and 50 mM NaCl by an Amicon centrifugal filter unit (Millipore). Prior to crystallization, the 6xHis tag was removed by overnight TEV protease digestion at room temperature. To remove the cleaved 6xHis fragments and the used TEV protease, the mixture was again transferred to a Ni-NTA agarose (Protino) column. The flow-through fraction was concentrated and buffer exchanged into 20 mM Tris-HCl (pH 8.0) and 50 mM NaCl by an Amicon centrifugal filter unit (Millipore) to obtain the protein with a concentration of at least 8 mg/mL. Protein purity was assessed using SDS-PAGE (S1 Fig).

### Crystallization, data collection, and structure determination

The crystallization was set by a Tecan Freedom EVO100 workstation (Tecan Group) in 96-well sitting-drop plates by combining 0.4 μL of protein with 0.4 μL of precipitant solution from JCSG-*plus* and Structure screen 1&2 sparse matrix screens (Molecular Dimensions). Only for FtlA_27-297_ were small needle-shaped crystals obtained from Structure screen 1&2 precipitant solution containing 2% dioxane, 0.1 M Bicine (pH 9.0), and 10% PEG 20 000. After optimization of the conditions, the crystals used for diffraction data collection were harvested from a solution containing 0.1 M Bicine (pH 9.0) and 6% PEG 20 000. Before data collection, the crystals were harvested using 25% glycerol as a cryoprotectant and stored in liquid nitrogen. The X-ray diffraction data measurements were carried out at the MX beamline instrument BL 14.1 at Helmholtz-Zentrum, Berlin [30]. The crystal structure of FtlA was determined using the single-wavelength anomalous dispersion (SAD) method. Reflections were indexed by XDS and scaled by AIMLESS from the CCP4 suite [31, 32]. The phases for FtlA were determined by SHELX C/D/E followed by model building in BUCCANEER [33, 34]. Manual model building and improvement was carried out in COOT [35]. Crystal structure refinement was performed with REFMAC5 [36]. A summary of the data collection, refinement, and validation statistics for FtlA is given in S1 Table.

### Structure prediction using AlphaFold

AlphaFold v2.0 [37] was used to predict the 3D structures for PFam12 members. Structure prediction with AlphaFold v2.0 was performed according to the default parameters as indicated at the website https://github.com/deepmind/alphafold/d/ running on AMD Ryzen Threadripper 2990 WX 32-Core; 128 GB RAM; 4 x NVIDIA GeForce RTX 2080, and using the full databases downloaded on 2022-09-25. For further structural analysis, only the predicted structure with the highest confidence was used (as ranked by using LDDT (pLDDT) scores).

### Site-directed mutagenesis

To mutate specific residues in FtlA into Ala, the corresponding nucleotides were replaced by using two complimentary primers (S2 Table) for PCR amplification of the pETm-11-bbk01 construct followed by *DpnI* endonuclease digestion and transformation in *E*. *coli* XL1-Blue cells as described above. The presence of the relevant mutation was confirmed by DNA sequencing.

### Electrophoretic Mobility Shift Assay (EMSA)

For the EMSA experiment to determine the double-stranded DNA (dsDNA) interaction with the PFam12 proteins (FtlA, FtlB, FtlC, FtlD and FtlE), a circular pETm-11 expression vector with a length of 5300 bp and three different PCR amplified products (coding regions *bb0236* with a length of 1938 bp, *bb0298* with a length of 636 bp, and *bba62* with a length of 141 bp) from the *B*. *burgdorferi* B31 genome were used with the intention of having DNA of different lengths, topologies and sequences.

All proteins were purified to equivalent purity as judged by SDS-PAGE followed by an identical dilution procedure where proteins were prepared as two-fold serial dilutions starting from 130 μM solution, while the DNA concentration for all samples was set to 45 nM as measured by NanoDrop. In total, five different PFam12 member protein dilutions were prepared, providing DNA-protein molar ratios from 1:180 to 1:2888. Five microliters of the respective dilution of the protein was mixed with 5 μL of respective DNA at a concentration of 45 nM and incubated for 15 min at room temperature. A 1% agarose gel containing ethidium bromide was run for 50 min at 110 mA in 1x TAE buffer. For the EMSA experiment with FtlA mutant proteins, the experiment was performed with only *bb0236* as a dsDNA sample. To determine the interaction with single-stranded DNA (ssDNA) a 73 nucleotide long forward primer corresponding to *B*. *burgdorferi bbp20* was used.

### Gel filtration chromatography

20 μL of sample protein (2 mg/mL) in 20 mM Tris-HCl (pH 8.0) and 50 mM sodium chloride was loaded on a prepacked Superdex 200 Increase 3.2/300 column (Cytiva) prepared with 150 mM phosphate buffer (pH 7.3) and connected to Shimadzu Prominence high-performance liquid chromatography system with diode array detection (Shimadzu). The flow rate was set to 0.1 mL/min. Ovalbumin (43 kDa) and chymotrypsinogen A (25 kDa) were used as MW reference standards.

### Isothermal titration calorimetry

Isothermal titration calorimetry (ITC) experiments were carried out on the MicroCal™ iTC200 instrument (Malvern Panalytical). Experiments were performed in 50 mM Tris-HCl (pH 7.5) and 50 mM NaCl buffer. All titrations were performed at 25°C using 70–120 μM protein. 280 μL of the protein solution in the sample cell were titrated with the dsDNA solution diluted to 35–50 μM depending on the protein concentration (to achieve a 2:1 ratio). The concentrations of proteins and dsDNA were determined by absorbance on a NanoDrop 2000c UV–Vis spectrometer (Thermo Fisher Scientific). Each titration started with a small 0.3 μL injection introduced to compensate the diffusion effects followed by 20×2.0 μL injections with 120 s time spacing between each injection. The steering speed was set to 600 rpm and the reference cell was filled with Milli Q water (Merck). The reference baseline consisting of small peaks of identical size (titration of the corresponding dsDNA into the buffer) was subtracted from the data before fitting to correct for the heat of the dsDNA dilution. ITC data were analyzed using Origin 7 SR4 (OriginLab). The first small-volume data point (used to compensate effects of titrant diffusion across the syringe tip during the equilibration process) was always removed prior to data fitting. “One Set of Sites” fitting model was applied.

### Circular dichroism

Circular dichroism (CD) measurements for the wild type and mutant proteins were performed on a Jasco J-1500 spectropolarimeter (Jasco). CD was performed to detect the secondary structure of different variants with the following parameters: continuous scanning mode with scanning speed of 50 nm/min, band width 0.1 nm, wave range 200–250 nm, data pitch 0.1 nm at 20°C. The spectra for 9 μM protein samples in 15 mM Tris-HCl (pH 8.0), 50 mM sodium chloride and 10 mM monosodium phosphate were recorded using a 2.0 mm light path length quartz cuvette.

## Results and discussion

### Crystal structure of FtlA

**Fig 1:**
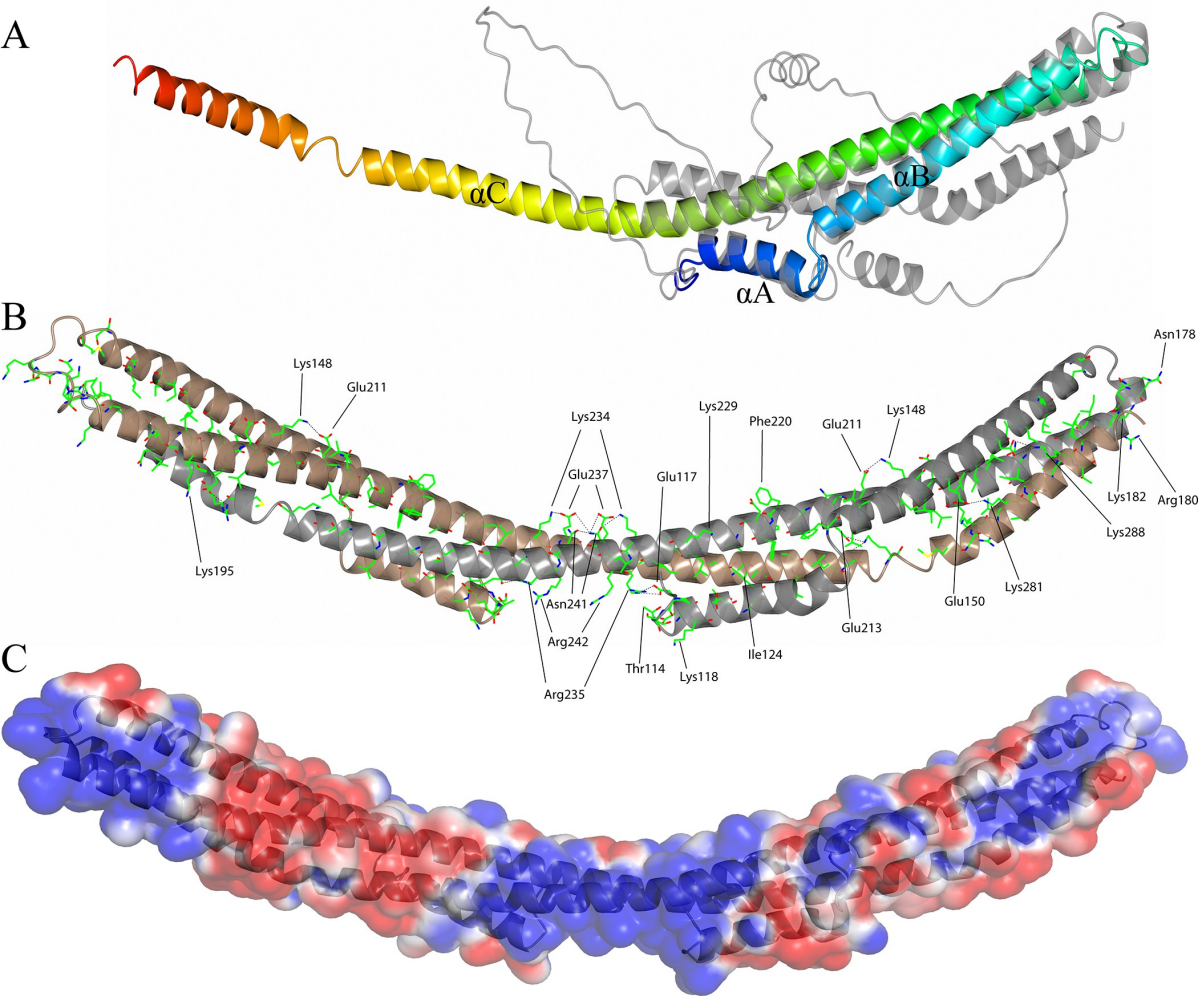
(A) The overall crystal structure of *B*. *burgdorferi* FtlA (blue at the N-terminus gradually switching to red at the C-terminus) superimposed with the predicted structure of FtlA (gray; C^α^ root-mean-square deviation 1.14 Ǻ). All three α-helices in the crystal structure have been designated αA to αC. (B) FtlA homodimer as observed in the crystal structure. In the homodimer, one FtlA molecule is illustrated in gray, while the second is shown in brown. The residues found to be conserved between the PFam12 members FtlA, FtlB, FtlC, FtlD, and FtlE are indicated. Ionic interactions between the side chains are shown as dotted lines. (C) Electrostatic surface potential of *B*. *burgdorferi* FtlA. The electrostatic potentials (red, negative; blue, positive) were calculated using APBS [39]. The surface contour levels were set to -1 kT/e (red) and +1 kT/e (blue).

The first 17 N-terminal residues followed by the Cys residue in FtlA form a putative signal sequence necessary for the covalent attachment of the protein to the membrane, where Cys18 becomes the first N-terminal residue after cleavage of the signal sequence. Therefore, the recombinant protein was expressed for residues 27–297 plus an additional N-terminal 6xHis tag separated by a *Tobacco Etch Virus* (TEV) cleavage site so that the 6xHis tag could be removed prior to crystallization. However, in the crystal structure of FtlA, only residues 110–297 were built in the final model, as electron density was not observed for N-terminal residues 28–109. The assumption that approximately the first 80 N-terminal residues in FtlA are disordered was approved by the AlphaFold [37] predicted model; therefore, the flexible nature of the N-terminal residues can explain their absence in the crystal structure of FtlA (Fig 1A). Most likely, the N-terminal flexible residues serve as a linker between the structural domain and the outer membrane as has been observed for other *B*. *burgdorferi* surface proteins [38]. In any case, the loss of the N-terminal residues does not affect the ability to bind DNA, as the PFam12 truncation mutants FtlA_110-297_, FtlB_110-297_, FtlC_131-312_, FtlD_126-306_ and FtlE_95-305_ were able to bind DNA equally well, as discussed later in more detail.

The protein crystals of FtlA diffracted to 2.7 Å resolution, and the crystal structure was determined by a single-wavelength anomalous diffraction (SAD) technique. The space group was C2 with two protomers per asymmetric unit. The overall monomer structure of FtlA consists of two comparatively short α-helices, αA and αB, and a 120 residue long helix αC, which are connected by short loop regions (Fig 1A).

In the protein crystal, FtlA forms a homodimer where the αC from one of the monomers interacts with the same α-helix in the other monomer by forming an antiparallel coiled coil structure of approximately 180 Ǻ in length (Fig 1B). To a lesser extent, αA and αB are also involved in the homodimer interface, resulting in a bundle of three α-helices at both ends of the homodimer. As the dimer interface is made by residues found on all three α-helices by interacting with the equivalent residues on the other monomer, the homodimer forms a massive 7950 Ǻ^2^ buried surface area as detected from PISA assembly analysis [40] and involves more than 50 residues. Between the monomers, the ionic interaction and the hydrophobic force dominate to form a coiled coil homodimer (Fig 1B). In comparison to the AlphaFold predicted structure, one essential difference is that the αC in the predicted structure does not form an extended α-helix but is bent backward and occupies exactly the same position as the other protomer in the crystal structure when it forms a dimer (Fig 1A and 1B).

DALI and PDBeFold analysis [41, 42] shows that the overall fold of FtlA indicates some structural similarity with several proteins (Table 1).

**Table 1:** Proteins with a similar fold to BBK01, as identified by DALI analysis.

No.	Protein	Organism	PDB ID	Z score	RMSD (Å)	Identity (%)	N residues
1.	Structural maintenance of chromosomes protein 2 (SMC)	*Saccharomyces cerevisiae*	4rsi-A	9.2	3.49	13	191
2.	Chromosome partition protein (SMC) (residues 463–719)	*Geobacillus stearothermophilus 10*	5h69-A	9.2	2.29	4	81
3.	Chromosome partition protein (SMC)	*Pyrococcus furiosus*	4rsj-A	9.0	3.92	8	106
4.	DNA double-strand break repair Rad50 ATPase (SMC)	*Pyrococcus furiosus*	1l8d-A	10.4	2.94	12	90
5.	Invasin IpaB	*Shigella flexneri*	5wkq-A	10.3	1.61	10	77
6.	Cytoplasmic dynein 1 heavy chain 1 (residues 3207–3483)	*Mus musculus*	3wuq-A	10.3	2.53	10	91
7.	Charged multivesicular body protein 4b (residues 23–97)	*Homo sapiens*	4abm-A	10.2	1.85	10	76
8.	Prefoldin subunit beta	*Pyrococcus horikoshii*	2zdi-B	10.1	1.54	7	46
9.	Prefoldin subunit beta	*Methanothermobacter thermautotrophicus*	1fxk-B	9.8	1.93	8	47
10.	Chromatin modification-related protein YNG2	*Saccharomyces cerevisiae*	5j9q-D	9.4	3.55	6	56
11.	Lysine-specific histone demethylase 1A	*Homo sapiens*	2x0l-A	10.7	3.98	9	118
12.	Membrane fusion protein (MFP) family protein	*Serratia marcescens*	5nen-A	10.8	3.77	5	112
13.	Centrosomal protein of 57 kDa	*Homo sapiens*	4l0r-A	10.7	1.54	7	37

**Fig 2:**
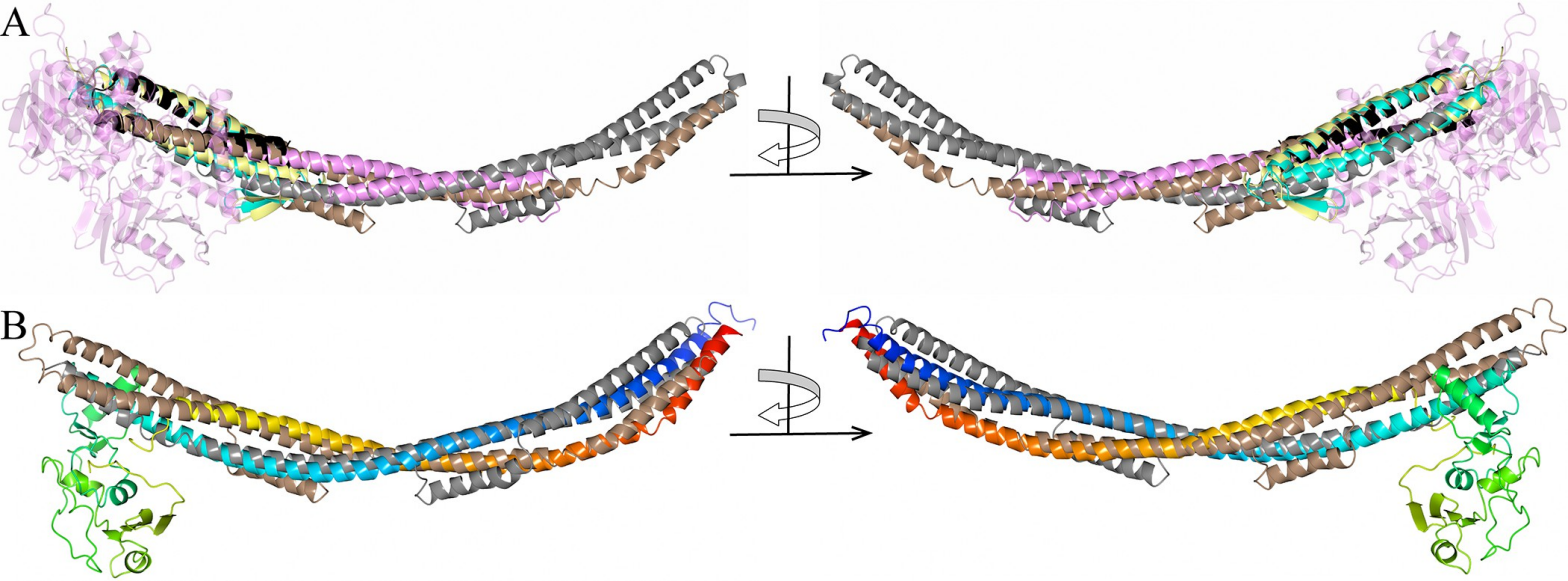
Crystal structure of *B*. *burgdorferi* FtlA superimposed with structurally similar proteins. (A) *B*. *burgdorferi* FtlA (gray, PDB ID 8CQN) superimposed with *H*. *sapiens* lysine-specific histone demethylase 1A (pink, the N- and C-terminal segments that do not overlap with FtlA are illustrated as slightly transparent, PDB ID 2X0L), *H*. *sapiens* charged multivesicular body protein 4b (black, PDB ID 4ABM), *P*. *horikoshii* prefoldin subunit beta (cyan, PDB ID 2ZDI) and *M*. *Thermautotrophicus* prefoldin subunit beta (yellow, PDB ID 1FXK). (B) *B*. *burgdorferi* FtlA superimposed with *S*. *cerevisiae* structural maintenance of chromosomes protein 2 (blend through model color scheme with blue at the N-terminus gradually switching to red at the C-terminus, PDB ID 4RSI).

For some of the proteins found by DALI, only individual α-helices or relatively small parts of the proteins are covered. For example, the *H*. *sapiens* lysine-specific histone demethylase 1A C-terminal domain shares some structural similarity with FtlA αA in both homodimer molecules, although the other segments in both proteins are not similar (Fig 2A).

Likewise, *H*. *sapiens* charged multivesicular body protein and prefoldin subunit beta from *P*. *horikoshii* and *M*. *thermautotrophicus* share conformational similarity only with FtlA αB and the N-terminus of αC (Fig 2A). In contrast, very good structural compatibility can be observed between FtlA and proteins that are members of the structural maintenance of chromosomes (SMC) protein family. The main members of the SMC family are condensin and cohesion, which are essential for chromosome localization and dynamics during DNA replication and segregation [43]. The SMC proteins are usually approximately 1000 residues long, where at the N-terminus, there is an ATP binding “head” domain followed by a coiled coil region, the “hinge” dimerization domain and again a coiled coil region and ATP binding “head” domain at the C-terminus [44–46]. The coiled coil regions are self-folded in an antiparallel fashion, producing an approximately 500 Ǻ long extended structure at one end of which there is an ATP binding domain but a dimerization domain at the other end [45]. By interacting via the dimerization domains, SMC proteins form a V-shaped dimer, and while in most bacteria, there is a single SMC protein that forms a homodimer, in eukaryotes, several SMC proteins are found that can form heterodimers [44]. It has been indicated that SMC proteins interact with DNA via the “hinge” domain near the coiled coil structural domain [46, 47]. The structural similarity for FtlA is found within the central coiled coil subunit of SMC proteins, as found by superimposing FtlA with SMC proteins from *S*. *cerevisiae*, *G*. *Stearothermophilus* and *Pyrococcus furiosus* (Fig 2B). The respective SMC crystal structures found by DALI are represented by truncated proteins, in which the “hinge” domain is followed by a part of the coiled coil domain. For example, in the *S*. *cerevisiae* SMC protein, 127 out of 297 coiled coil domain residues have been produced for the crystallized protein, while in *G*. *stearothermophilus*, only 38 out of 322 residues have been produced, but in *P*. *furiosus*, 59 out of 339 residues have been produced. Since the coiled coil region in the crystallized SMC protein from *S*. *cerevisiae* was by far the longest, it was used for structural alignment (Fig 2B). However, we expect that the other two SMC proteins would superimpose in a similar manner for the entire length of FtlA if they had longer coiled coil regions in their crystal structures, which was also indicated by the protein models predicted by AlphaFold. As seen from *B*. *burgdorferi* FtlA and *S*. *cerevisiae* SMC protein superimposed structures, both αC helices of the FtlA homodimer are structurally analogous to the coiled coil domain of the SMC protein. FtlA lacks any further structural domains, such as the “hinge” domain in the SMC protein, whereas in the SMC protein, there are no additional α-helices that would match αA and αB in FtlA.

### *B*. *burgdorferi* paralogous gene family 12

**Fig 3:**
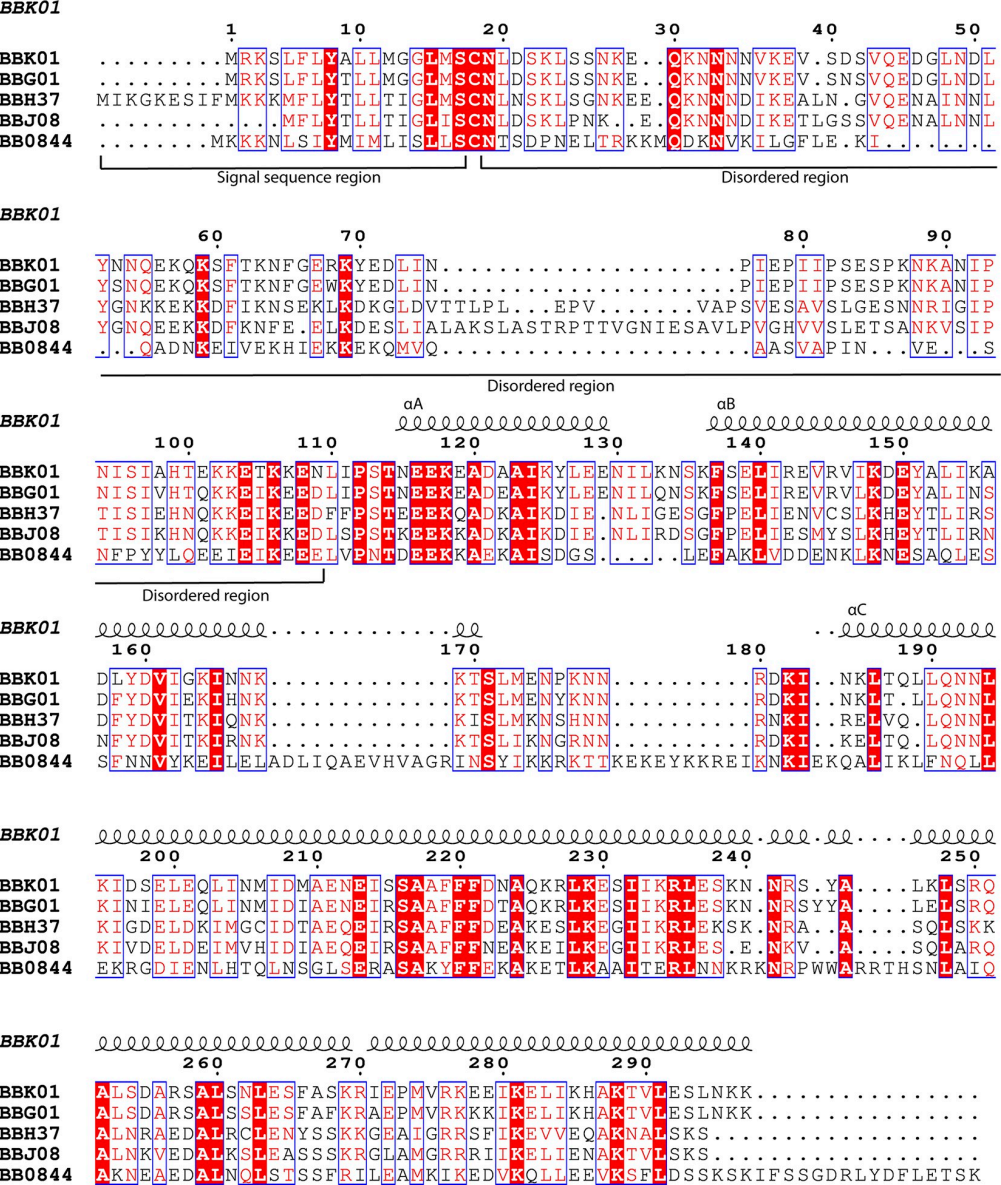
The sequence alignment of FtlA, FtlB, FtlC, FtlD and FtlE (BBK01, BBG01, BBH37, BBJ08, and BB0844 accordingly) was made by using the *Clustal Omega* multiple sequence alignment tool followed by ESPript3 [49, 50]. Conserved residues are illustrated with a red background, while conserved substitutions are illustrated in red and framed. Numbering and secondary structure elements with indicated αA, αB or αC above the alignment are shown for FtlA. Approximate locations of the signal sequences and disordered N-terminal residues for the PFam12 members are indicated below the sequence alignment.

The genome of *B*. *burgdorferi* B31 is composed of a 900 kb linear chromosome and 12 linear and 9 circular plasmids of different sizes, in total approximately 600 kb [13, 48]. *B*. *burgdorferi* B31 PFam12 contains five paralogous proteins–FtlA, FtlB, FtlC, FtlD and FtlE (genes *bbk01*, *bbg01*, *bbh37*, *bbj08*, and *bb0844* accordingly) distributed over a chromosome and linear plasmids (lp) lp28-2, lp28-3, lp38 and lp36 accordingly. Sequence alignment showed that the PFam12 members share 31% to 69% identity (Fig 3).

Once the conserved residues, as found from the sequence alignment of the PFam12 members, are presented on the crystal structure of FtlA, it can be seen that the majority of the residues are necessary for proper positioning of the three α-helices or are involved in the formation of the dimer interface (Fig 1B) and include both hydrophobic and charged interactions, typical for maintenance of fold in coiled coil proteins. For example, Lys148, Glu211, Lys281, Glu150, Asn241, Glu237, and Lys234 are most likely needed to maintain the correct tertiary structure and to stabilize the homodimer through interactions between the coils, as they are involved in ionic and hydrogen bond formation between different segments (Fig 1B). In particular, the residues Asn241, Glu237, and Lys234 should be noted, as in the middle of the homodimer, these residues form an extensive mutual interaction to stabilize the central part of the dimer. However, for several conserved charged residues, especially Arg242, Arg235, and Lys118, which are located in a pocket-like cavity, there is no provisional influence on fold stabilization or dimer formation and could be thus related to protein function.

As judged from the sequence alignment, the greatest differences from the other members are shown by FtlE, where several insertions are present at the end of αB and in the following loop region, as well as at the N-terminal end of the protein (Fig 3).

By using the NCBI BLAST search [51] against the *Borreliaceae* family, PFam12 members were found to be present in 8 different *Borrelia* species, including the main Lyme disease causing spirochetes (Table 2).

**Table 2:** Presence of PFam12 members in different *Borrelia* species.

Protein *Borrelia* species	FtlA	FtlB	FtlC	FtlD	FtlE
*B*. *burgdorferi* B31	+ (100/100)	+ (100/100)	+ (100/100)	+ (100/100)	+ (100/100)
*B*. *garinii* PBr	+ (97/68)	+ (97/69)	+ (97/81)	+ (100/73)	+ (75/52)
*B*. *afzelii* PKo	+ (100/75)	+ (100/74)	-	+ (99/50)	+ (95/61)
*B*. *bavariensis* PBi	+ (100/75)	+ (100/74)	+ (97/83)	+ (100/73)	+ (98/65)
*B*. *mayonii* MN14-1420	+ (100/94)	+ (100/90)	-	+ (99/54)	-
*B*. *valaisiana* VS116	-	-	+ (97/59)	+ (100/63)	-
*B*. *finlandesis* SV1	+ (100/68)	+ (100/69)	-	-	-
*B*. *bissettii* DN127	+ (100/86)	+ (100/85)	-	-	+ (87/48)

The sequence coverage/identity is indicated in parentheses.

The *B*. *burgdorferi* PFam12 members are lipoproteins, as they contain an N-terminal signal peptide for translocation of the protein across the cytoplasmic membrane and lipidation, as can be predicted by SignalP 4.1 and previously confirmed experimentally [13, 24, 28]. However, after covalent modification by a fatty acid at its N-terminal Cys residue, the protein can be targeted into the periplasmic side of the cytoplasmic membrane, the periplasmic side of the outer membrane, or the external side of the outer membrane [12]. To clarify the exact location of the PFam12 proteins, prior experimental evidence indicated that FtlE is most likely a periplasmic inner membrane-attached protein, while FtlA, FtlB and FtlCare surface-exposed lipoproteins [23, 24, 27]. Since the lipoprotein signal sequence region in spirochetes may exhibit comparatively high variability, the PFam12 member FtlD was not included in any of the previous studies for lipoprotein localization [12]. Although the signal peptide score for FtlD by SignalP 4.1 is indeed relatively low compared to the other PFam12 members, at the N-terminus of FtlD, a spirochetal lipobox can be identified (residues GLISC) [12, 28]. The presence of a lipoprotein signal peptide in FtlD was also confirmed by three different signal peptide prediction tools–Phobius (prediction of transmembrane topology and signal peptides), TMpred, and TOPCONS (consensus prediction of membrane protein topology and signal peptides) [52, 53]. Thus, FtlD can be considered a lipoprotein, but there are no experimental data for the exact localization of FtlD either in the periplasm as for FtlE or the outer surface as for FtlC, FtlB, and FtlA.

Since *B*. *burgdorferi* spends its life either in *Ixodes* ticks or a mammalian host, much has been studied about the changes in the gene expression pattern depending on the environmental conditions [8, 14, 15]. Regarding PFam12 members, by using dialysis membrane chambers (DMCs) implanted in rats and thus simulating mammalian host conditions, it has been demonstrated that *ftlE* is highly upregulated [14, 25]. Meanwhile, the influence of a pH change from pH 8.0 to pH 7.0 simulating the change experienced by spirochetes during the transfer from ticks to mammalian hosts caused significant upregulation of *ftlA* [26]. In turn, the influence of temperature on gene expression in *B*. *burgdorferi* grown at 23°C and 35°C showed significantly greater levels of expression at 35°C for *ftlE*, *ftlC*, and *ftlA*, while *ftlD* indicated higher expression levels at 23°C than at 35°C [8]. Overall, this clearly indicates that most PFam12 members tend to be upregulated as the tick starts its blood meal and the spirochetes enter the new host.

### DNA binding

**Fig 4:**
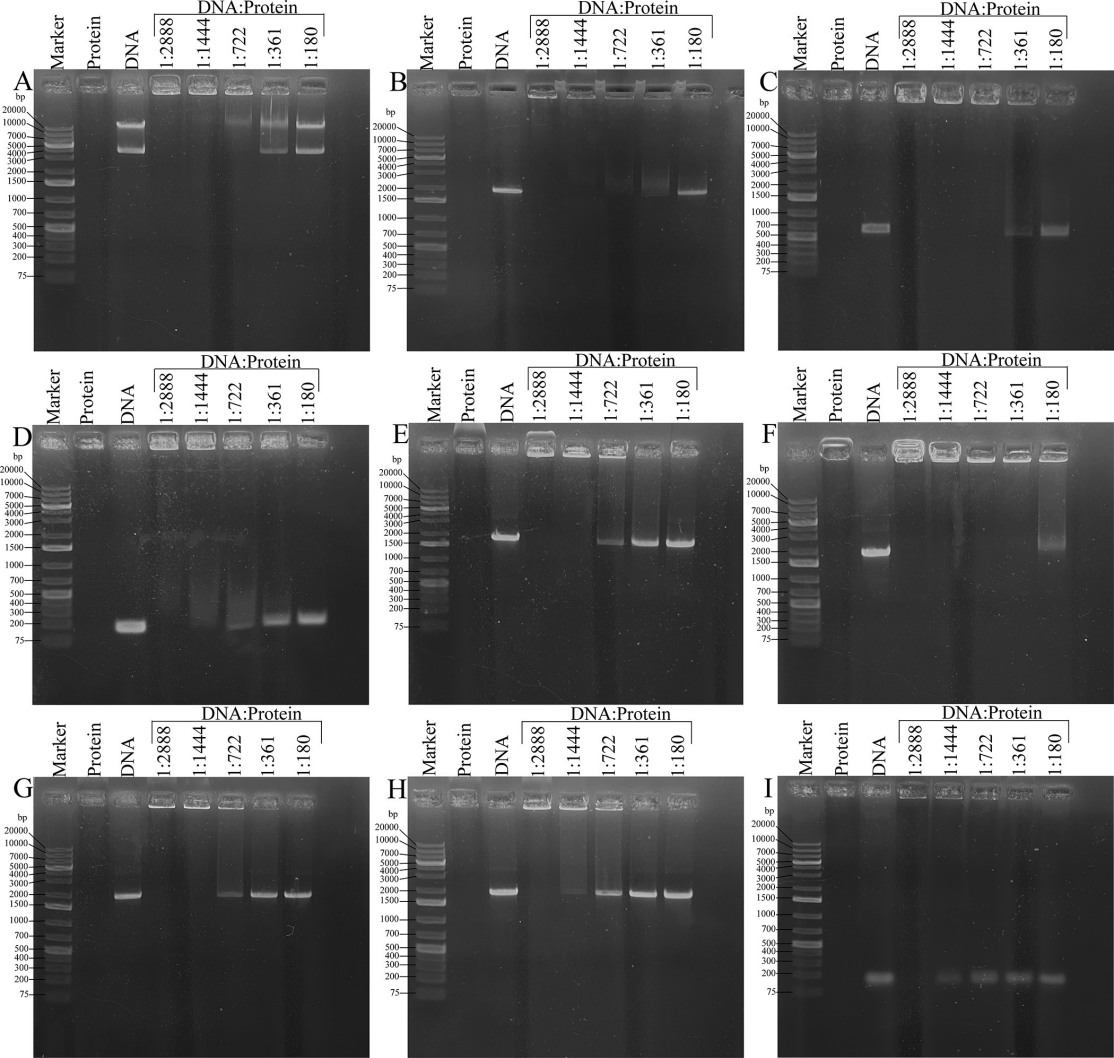
Agarose gel electrophoresis mobility shift assay. The assay with FtlA was performed using four unrelated dsDNA fragments that additionally differed in length and topology: (A) a circular pETm-11 expression vector of 5300 bp; (B) *bb0236* of 1938 bp; (C) *bb0298* of 636 bp, and (D) *bba62* of 141 bp. For FtlB, FtlC, FtlD, and FtlE, the experiment with 1938 bp DNA fragment is presented (E-H). The DNA:protein molar ratios ranged from 1:2888 to 1:180. (I) EMSA for FtlA with 73 nucleotide long ssDNA fragment. The sizes of DNA ladder fragments, ranging from 75 bp to 20000 bp, are indicated.

After expression in *E*. *coli*, with the following cell lysis, centrifugation, and purification by affinity chromatography, PFam12 members (FtlA, FtlB, FtlC, FtlD, and FtlE) had a UV absorption maximum at 260 nm instead of absorption at 280 nm that is typical for proteins. Taking into account the characteristic absorption of nucleic acids at 260 nm, and the structural similarity of these proteins to DNA-binding proteins, a potential protein-DNA interaction was suspected. Therefore, interaction with diverse DNA fragments of different lengths and topologies with PFam12 member proteins was analyzed in an agarose gel electrophoresis mobility shift assay (EMSA). A distinctive shift of DNA was observed for all the PFam12 members and for all the different dsDNA fragments that were used, while a relatively weak interaction was found with ssDNA (Fig 4).

Thus, the EMSA experiments confirmed the assumption that PFam12 member proteins are able to bind DNA. Since based on the structural analysis, the first 100–120 N-terminal residues are disordered, for the DNA-binding experiments, not only full-length proteins excluding the signal sequence region but also the N-terminally truncated PFam12 proteins FtlA_110-297_, FtlB_110-297_, FtlC_131-312_, FtlD_126-306_, and FtlE_95-323_ were used (Fig 3). Based on the EMSA experiment showing protein-DNA interaction with N-terminally truncated proteins (Fig 4), it suggests that the N-terminal segment most likely does not have a direct role in DNA binding.

**Fig 5:**
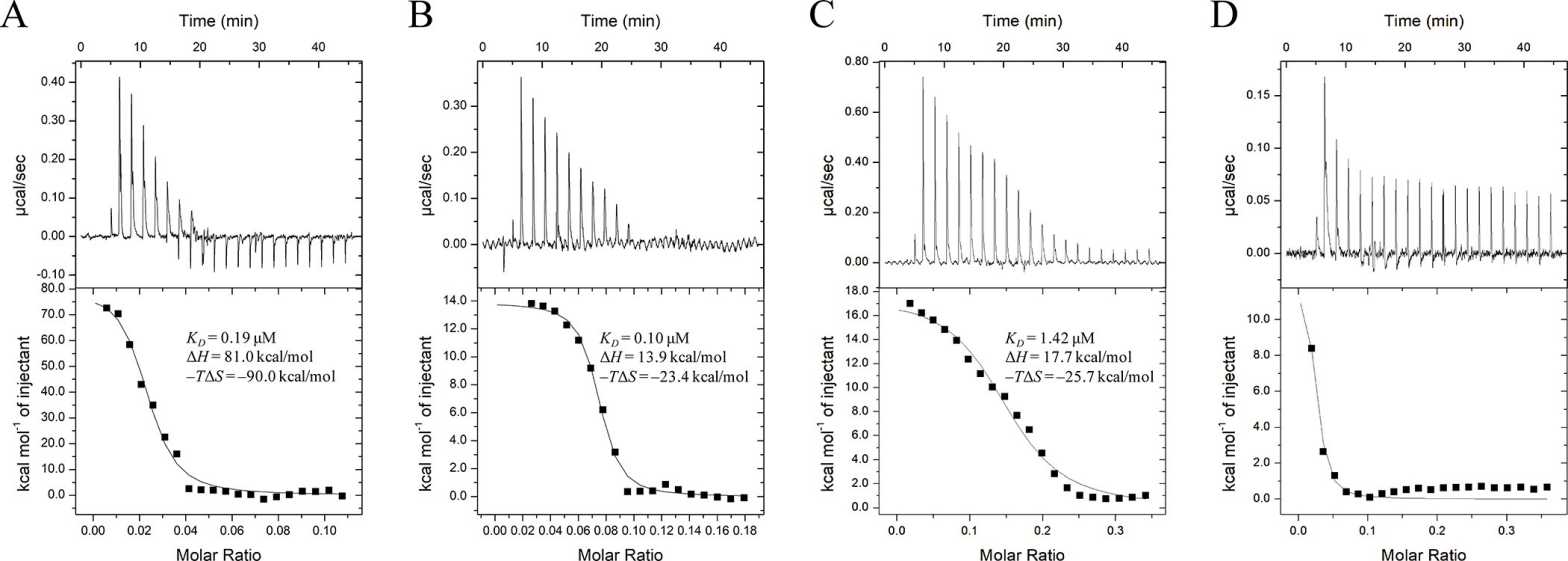
ITC titration of FtlA with dsDNA of varying nucleotide length. (A) ITC titration data for FtlA with 78 bp DNA, (B) 36 bp DNA, and (C) 24 bp DNA. (D) Titration data for the FtlA Arg242Ala + Arg180Ala mutant protein with dsDNA. The upper portion of the figures shows the amount of heat produced per injection, while the lower portion shows the amount of heat generated per injection as a function of the concentration of the corresponding dsDNA. The smooth line represents the best fit of the experimental data to the one-site model.

To characterize the protein-DNA interaction in more detail, we performed an ITC experiment with FtlA_110-297_ using three different dsDNA fragments of varying sequence and length (Fig 5 and S3 Table). It is worth mentioning that all titration experiments were performed with a non-standard 2:1 protein-ligand ratio. This ratio was found to be the most optimal for achieving an S-shaped titration curve. Using the commonly used protein-ligand ratio of 1:10 did not provide any meaningful heat effects, indicating full protein saturation by the dsDNA form the very beginning. The stoichiometry obtained in these titration experiments varied within 0.02 and 0.15, indicating the simultaneously binding of multiple protein molecules to a single dsDNA molecule. Based on the structural data, it is likely that FtlA forms a homodimer for DNA interaction. Since the interaction is non-specific, multiple dimeric molecules can potentially bind to a single DNA molecule. Thermodynamic parameters were similar between the different dsDNA samples (*K*_*D*_ = 0.19 μM, 0.10 μM and 1.42 μM for 78 bp, 36 bp and 24 bp DNA accordingly). No interaction with DNA was observed for the FtlA_110-297_ Arg242Ala + Arg180Ala double mutant (Fig 5D), consistent with the EMSA experiment described below.

**Fig 6:**
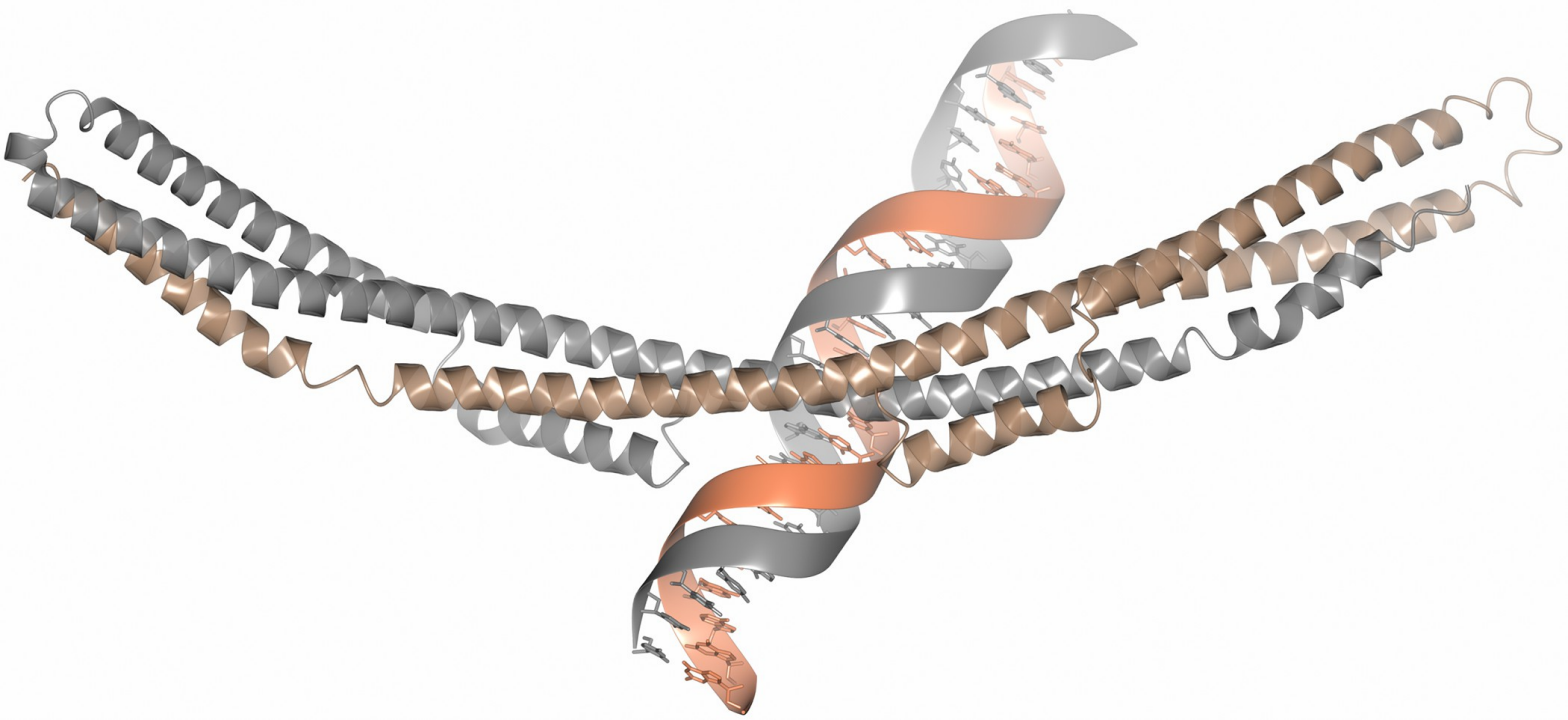
Molecular docking of FtlA with the 36 bp DNA fragment. In the homodimer, one FtlA molecule is illustrated in gray, while the second is shown in brown.

As judged from the crystal structure of FtlA and taking into account the conserved residues of the PFam12 members, the potential DNA-binding site in the PFam12 proteins might be located in the middle of the dimer, along residues Arg242, Arg235, and Lys118 or the distal ends of the dimer where conserved residues Lys281 and Lys182 are located (Fig 1B). This assumption is supported by the electrostatic surface potential map, which shows a distinctive positive charge at the middle segment of FtlA and at both ends of the dimer (Fig 1C). Additionally, the interaction between FtlA and 36 bp DNA was studied through docking analysis using the HDOCK server with default paramaters [54]. The analysis produced a protein-DNA complex with a docking score of -247.85 and a confidence score of 0.87 (a docking score lower that -200 and a confidence score above 0.7 are considered indicative of a high-quality model) (Fig 6). These results confirm our assumption, based on conserved residues and electrostatic potentials, that the most likely DNA binding site is located in the central region of the dimer.

Although FtlA shares structural similarities with SMC proteins, which also function as DNA-binding proteins, the binding pattern must clearly be different. In SMC proteins, the DNA-binding site has been proposed to be located at the dimerization domain, and the coiled coil region connects the dimerization and ATP binding domains [46, 47]. In contrast, FtlA does not possess any known DNA binding or dimerization domains, although FtlA has additional α-helices A and B (Fig 1B).

### Residues involved in DNA binding

**Fig 7:**
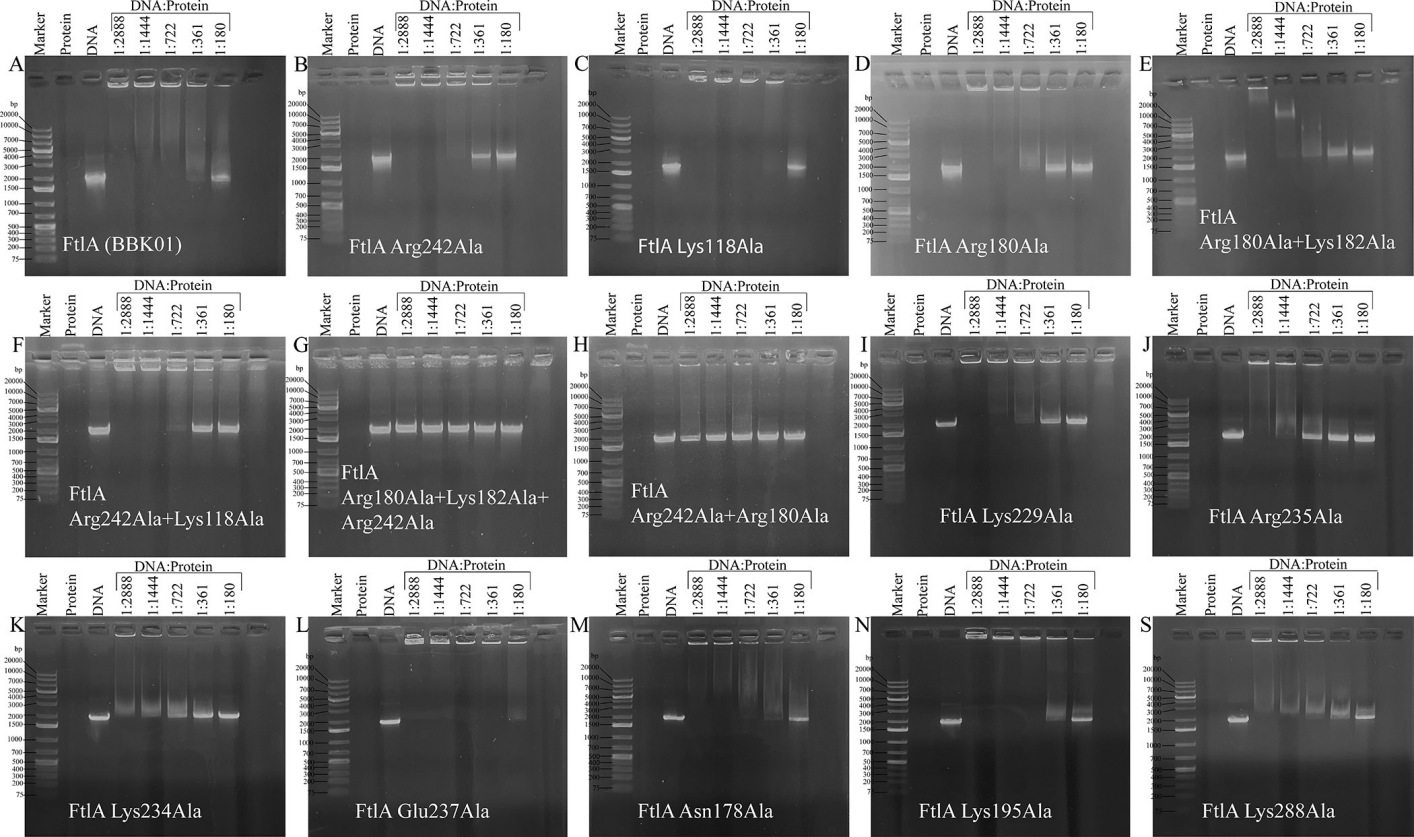
Agarose gel electrophoresis mobility shift assay showing the protein and DNA interaction with wild-type FtlA (A) and all 14 mutants, including 10 single mutants (B, C, D, I, J, K, L, M, N, and S), 3 double mutants (E, F, and H), and 1 triple mutant (G). The sizes of DNA Ladder fragments ranging from 75 bp to 20000 bp are indicated.

To determine the role of specific residues in DNA binding, several PFam12 conserved residues found from sequence alignment (Fig 3) were mutated into Ala with particular attention given to the charged residues located on the surface. By using site-directed mutagenesis, a total of 14 mutants were generated, including 10 single mutants, 3 double mutants, and 1 triple mutant. From the mutant analysis, it was confirmed that the middle part of the dimer is involved in DNA binding as proposed above, but at the same time, the residues located at the ends of the dimer are also involved in DNA binding (Figs 1B and 7).

**Fig 8:**
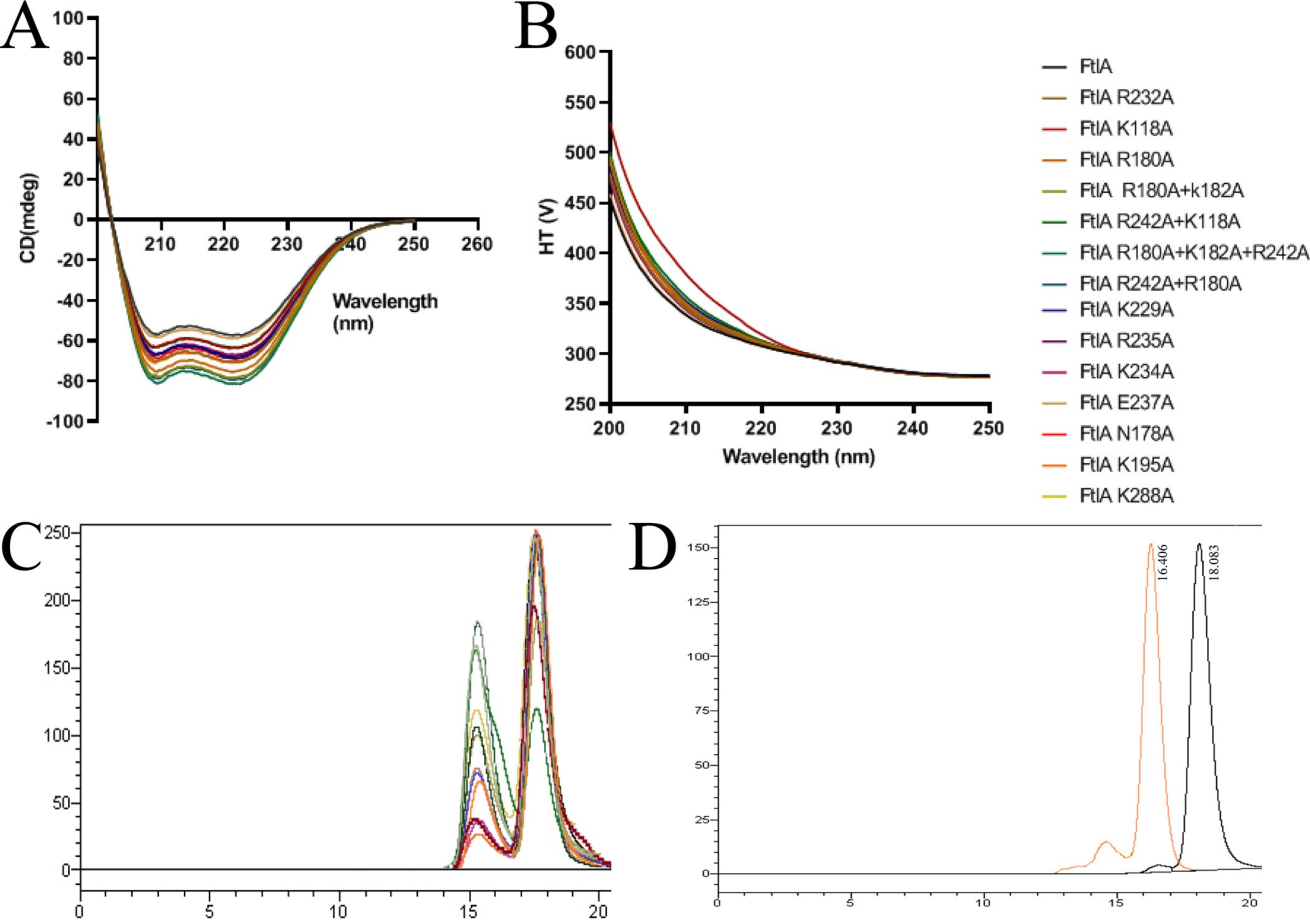
Protein secondary structure composition and oligomerization analysis. (A) An overlay of CD spectra of both wild-type FtlA and all 14 FtlA mutant proteins, showing a characteristic CD spectrum indicative of α-helical secondary structure, which is consistent across all samples. (B) The corresponding high tension voltage traces generated from the CD experiment. (C) Size-exclusion chromatography elution profiles for FltA and all 14 FtlA mutant proteins. (D) Size-exclusion chromatography elution profiles for ovalbumin (43 kDa, orange) and chymotrypsinogen A (25 kDa, black) used as MW reference standards.

In particular, the mutations of the conserved residues Arg242 and Arg235 located at the middle of the dimer and Arg180 and Lys182 located at the end of the dimer showed a reduced ability to interact with DNA, and a double mutant Arg242Ala + Arg180Ala showed a complete loss of DNA binding. Mutation of Glu237 showed even better interaction with DNA than wild-type FtlA, which could be explained by the fact that the negative charge that could interfere with DNA binding is removed. From mutagenesis studies it can be concluded that the interaction with the DNA involves a large part of the FtlA surface and is not localized to only the central part or the terminal parts of the dimer. To ensure that the introduced mutations did not affect protein folding, a CD spectroscopy was performed for both the wild type FtlA and the mutated proteins. The results showed that all of them exhibited similar CD spectra characteristic for α-helical secondary structure (Fig 8A). In turn, to understand whether the introduced mutations had any impact on protein oligomerization state a gel-filtration chromatography was performed. This analysis indicated a comparable oligomerization profile for both the native FtlA and all the mutant proteins. Under the respective gel filtration conditions, two peaks corresponding to monomer and dimer were observed (Fig 8C).

### Function of the PFam12 member proteins

Although previous studies have indicated that FtlE does not play any role in the pathogenesis of Lyme disease in a mouse-tick infection model with an introduced FtlE deletion mutant, the other PFam12 members were not taken into account, meaning that the other members could compensate for the lack of FtlE [55]. Considering that PFam12 members are lipoproteins located on the outer membrane (FtlA, FtlB and FtlC) or periplasm (FtlE) and are DNA-binding proteins, as one of the possible functions of PFam12 members could be associated with the formation of protective matrix or biofilm. Little is known about the interaction with eDNA, although it has been demonstrated that in *Staphylococcus aureus*, *Haemophilus influenza*, and *Streptococcus pneumoniae*, there are some proteins potentially binding to and stabilizing eDNA [56–58]. Although in a few studies it has been demonstrated that *B*. *burgdorferi*, *B*. *afzelii*, and *B*. *garinii* possess a biofilm made from polysaccharides and a significant amount of extracellular DNA (eDNA) [59–62] this assumption has not been generally accepted in the Lyme disease research community mainly because there are no conclusive in vivo studies that Lyme disease-causing spirochetes form a biofilm. At present, we can only speculate that another reason for eDNA binding might be to use eDNA as a source of nucleotides, particularly given that *B*. *burgdorferi* lacks a large portion of biosynthesis pathways, including nucleotide synthesis [48].

Although several DNA-binding proteins have been previously described in *B*. *burgdorferi*, these proteins were cytoplasmic and are involved in gene expression regulation [10]. Of course, this is not possible for PFam12 surface/periplasm localized lipoproteins, which also do not have a transmembrane part, suggesting that the proteins could most likely be related to sensing or gathering the eDNA although the exact role of PFam12 in *B*. *burgdorferi* remains to be determined.

## Conclusions

In the current study, we determined the crystal structure of the PFam12 member protein FtlA and revealed that the PFam12 member proteins FtlA, FtlB, FtlC, FtlD, and FtlE are non-specific DNA-binding proteins. The residues that play a role in DNA binding were identified. Considering the localization and DNA-binding ability of the PFam12 members, these proteins serve as intriguing targets for future functional studies. Overall, identifying the ligand of all PFam12 members and characterizing the mode of interaction is an important contribution to understand the complex nature of the Lyme disease agent.

## Supporting information

S1 Fig SDS-PAGE analysis of PFam12 members. (TIF)

S1 Table Statistics for data and structure quality. (DOC)

S2 Table Oligonucleotides used for site-directed mutagenesis. (DOC)

S3 Table Sequences of top strands of dsDNA used in ITC experiment. (DOCX)
