# Supplementary figures and images for: Members of the paralogous gene family 12 from the Lyme disease agent Borrelia burgdorferi are non-specific DNA-binding proteins
Source: PLoS One. 2024 Apr 16;19(4):e0296127. doi: 10.1371/journal.pone.0296127 (PMC11020477; doi:10.1371/journal.pone.0296127)

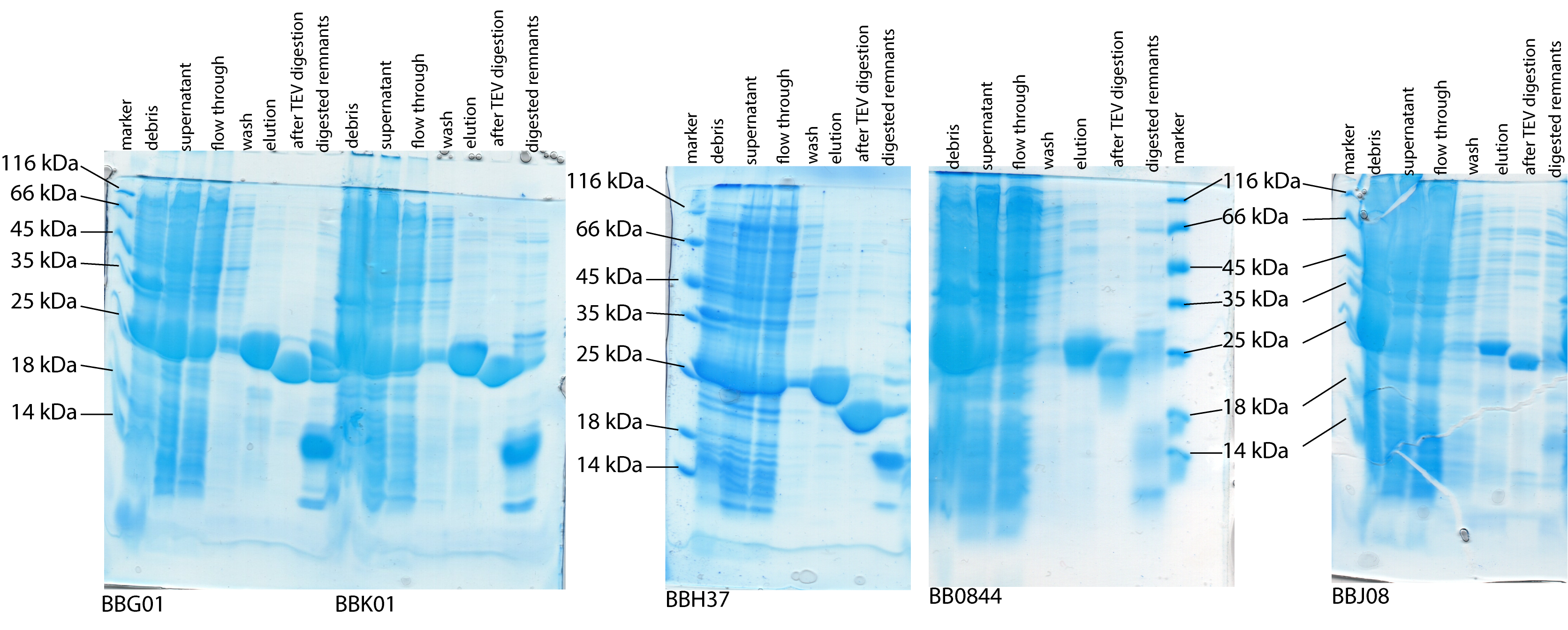

Supplement: S1 Fig — (TIF) [file pone.0296127.s001.tif]
