# Supplementary material for: Members of the paralogous gene family 12 from the Lyme disease agent Borrelia burgdorferi are non-specific DNA-binding proteins
Source: PLoS One. 2024 Apr 16;19(4):e0296127. doi: 10.1371/journal.pone.0296127 (PMC11020477; doi:10.1371/journal.pone.0296127)
Supplement: S1 Table — (DOC) [file pone.0296127.s002.doc]

Table S1. Statistics for Data and Structure Quality.

| **Dataset** | **BBK01 native** | | **BBK01 Se-Met** |
| --- | --- | --- | --- |
| **Space group** | C 2 | | C 2 |
| **Unit cell dimensions** |  | |  |
| **a (Å)** | 99.07 | | 99.44 |
| **b (Å)** | 52.13 | | 52.72 |
| **c (Å)** | 95.02 | | 94.99 |
| **Wavelength (Å)** | 0.9184 | | 0.9796 |
| **Resolution (Å)** | 46.14-2.70 | | 47.49-3.30 |
| **Highest resolution bin (Å)** | 2.83-2.70 | | 3.56-3.30 |
| **No. of reflections** | 45603 | | 42376 |
| **No. of unique reflections** | 13352 | | 7221 |
| **Completeness (%)** | 98.6 (98.7) | | 95.8 (94.1) |
| **R_merge_** | 0.05 (0.24) | | 0.09 (0.26) |
| ***I/σ* (*I*)** | 12.3 (4.0) | | 11.3 (6.3) |
| **Multiplicity** | 3.4 (3.4) | | 5.9 (5.5) |
| **Refinement** |  | |  |
| **R_work_** | 0.212 (0.257) | | 0.168 (0.161) |
| **R_free_** | 0.243 (0.287) | | 0.253 (0.245) |
| **Average B-factor (Å^2^)** |  | |  |
| **Overall** | 59.4 | | 59.7 |
| **From Wilson plot** | 52.8 | | 52.5 |
| **No. of atoms** |  | |  |
| **Protein** | 3004 | | 2917 |
| **Water** | 0 | | 0 |
| **RMS deviations from ideal** | |  |  |
| **Bond lengths (Å)** | 0.011 | | 0.008 |
| **Bond angles (^o^)** | 1.747 | | 1.508 |
| **Ramachandran outliers (%)** | |  |  |
| **Residues in most favored regions (%)** | 90.76 | | 78.87 |
| **Residues in allowed regions (%)** | 6.25 | | 15.50 |
| **Outliers (%)** | 2.99 | | 5.63 |

Values in parentheses are for the highest resolution bin.
