# Supplementary material for: Members of the paralogous gene family 12 from the Lyme disease agent Borrelia burgdorferi are non-specific DNA-binding proteins
Source: PLoS One. 2024 Apr 16;19(4):e0296127. doi: 10.1371/journal.pone.0296127 (PMC11020477; doi:10.1371/journal.pone.0296127)
Supplement: S2 Table — (DOC) [file pone.0296127.s003.doc]

**Table S2.** Oligonucleotides used for site-directed mutagenesis.

| Oligonucleotide | Sequence (5’-3’) |
| --- | --- |
| *Bbk01* Lys229Ala | CAG AAA AGG TTA GCA GAA AGC ATT ATT AAA AGA TTA GAG AGT and AAT AAT GCT TTC TGC TAA CCT TTT CTG AGC GTT GTC AAA AAA |
| *Bbk01* Arg235Ala | AGC ATT ATT AAA GCA TTA GAG AGT AAA AAT AAT AGA TCT TAT and TTT ACT CTC TAA TGC TTT AAT AAT GCT TTC TTT TAA CCT TTT |
| *Bbk01* Lys234Ala | GAA AGC ATT ATT GCA AGA TTA GAG AGT AAA AAT AAT AGA TCT and ACT CTC TAA TCT TGC AAT AAT GCT TTC TTT TAA CCT TTT CTG |
| *Bbk01* Glu237Ala | ATT AAA AGA TTA GCA AGT AAA AAT AAT AGA TCT TAT GCA TTA and ATT ATT TTT ACT TGC TAA TCT TTT AAT AAT GCT TTC TTT TAA |
| *Bbk01* Asn178Ala | GAG AAT CCT AAG GCA AAT AGA GAT AAG ATA AAT AAA TTA ACA and CTT ATC TCT ATT TGC CTT AGG ATT CTC CAT TAA TGA TGT TTT |
| *Bbk01* Lys195Ala | CAA AAT AAT TTA GCA ATA GAT AGT GAA CTT GAG CAG CTT ATA and TTC ACT ATC TAT TGC TAA ATT ATT TTG CAA CAA TTG TGT TAA |
| *Bbk01* Lys288Ala | ATT AAA CAT GCA GCA ACT GTT TTA GAA AGT CTC AAT AAA AAA and TTC TAA AAC AGT TGC TGC ATG TTT AAT AAG CTC TTT TAT TTC |
| *Bbk01* Arg242Ala | GAG AGT AAA AAT AAT GCA TCT TAT GCA TTA AAA and TTT TAA TGC ATA AGA TGC ATT ATT TTT ACT CTC |
| *Bbk01* Lys118Ala | TCT ACT AAT GAA GAA GCA GAA GCT GAT GCA GCA and TGC TGC ATC AGC TTC TGC TTC TTC ATT AGT AGA |
| *Bbk01* Arg180Ala | AAT CCT AAG AAC AAT GCA GAT AAG ATA AAT AAA and TTT ATT TAT CTT ATC TGC ATT GTT CTT AGG ATT |
| *Bbk01* Lys182Ala | AAG AAC AAT AGA GAT GCA ATA AAT AAA TTA ACA and TGT TAA TTT ATT TAT TGC ATC TCT ATT GTT CTT |
