# Supplementary material for: Members of the paralogous gene family 12 from the Lyme disease agent Borrelia burgdorferi are non-specific DNA-binding proteins
Source: PLoS One. 2024 Apr 16;19(4):e0296127. doi: 10.1371/journal.pone.0296127 (PMC11020477; doi:10.1371/journal.pone.0296127)
Supplement: S3 Table — (DOCX) [file pone.0296127.s004.docx]

**Table S3.** Sequences of top strands of dsDNA used in ITC experiment.

| dsDNA | Sequence (5’-3’) |
| --- | --- |
| Sample KB1001 (78 bp) | GAA TGG AGA AAT TTA AAA GAT CAA GGC TAT AAG GTT CCC TAT CTT AGA CAT TTG ATT TCT ACT ATT GAG CAA AGG AGA |
| Sample KB1003 (36 bp) | AGT TAT TTA AAA TCA CCT TAT GAT GTT ATT GAA GCT |
| Sample KB861 (24 bp) | AAG TTT GGC GAG GCT CTA GTA ACG |
